# Supplementary figures and images for: VCAM1/VLA4 interaction mediates Ly6Clow monocyte recruitment to the brain in a TNFR signaling dependent manner during fungal infection
Source: PLoS Pathog. 2020 Feb 26;16(2):e1008361. doi: 10.1371/journal.ppat.1008361 (PMC7062284; doi:10.1371/journal.ppat.1008361)

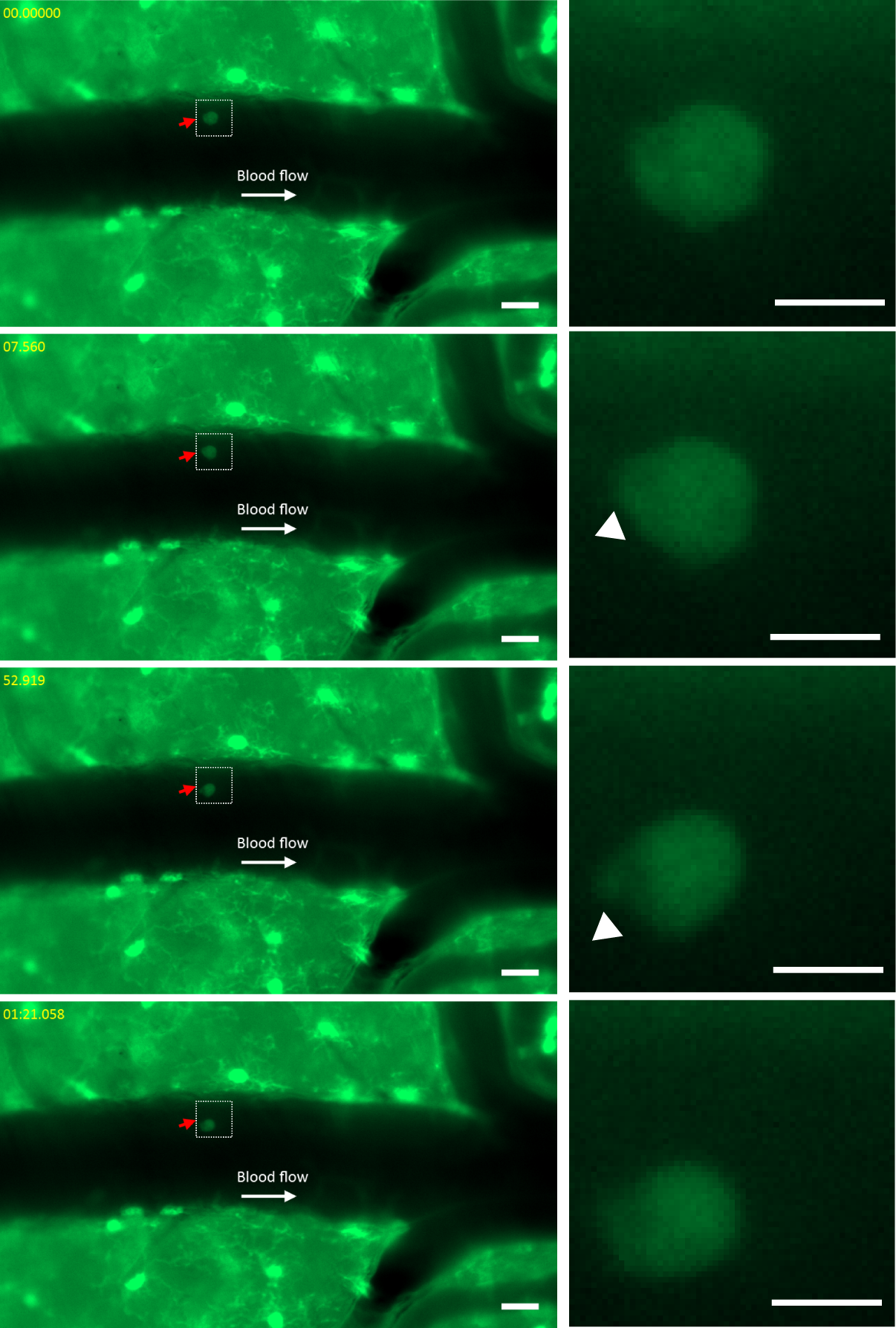

Supplement: S1 Fig — IVM was performed on the brain of a naïve CX3CR1gfp/+ mouse. A series of images showing a monocyte (red arrow) crawling against the blood flow along the postcapillary venule (left panel). The enlargement on the right panel showing the leading edge of the crawling monocyte (white arrowhead). See also S2 Video. Scale bars: 10 μm. (TIF) [file ppat.1008361.s001.tif]

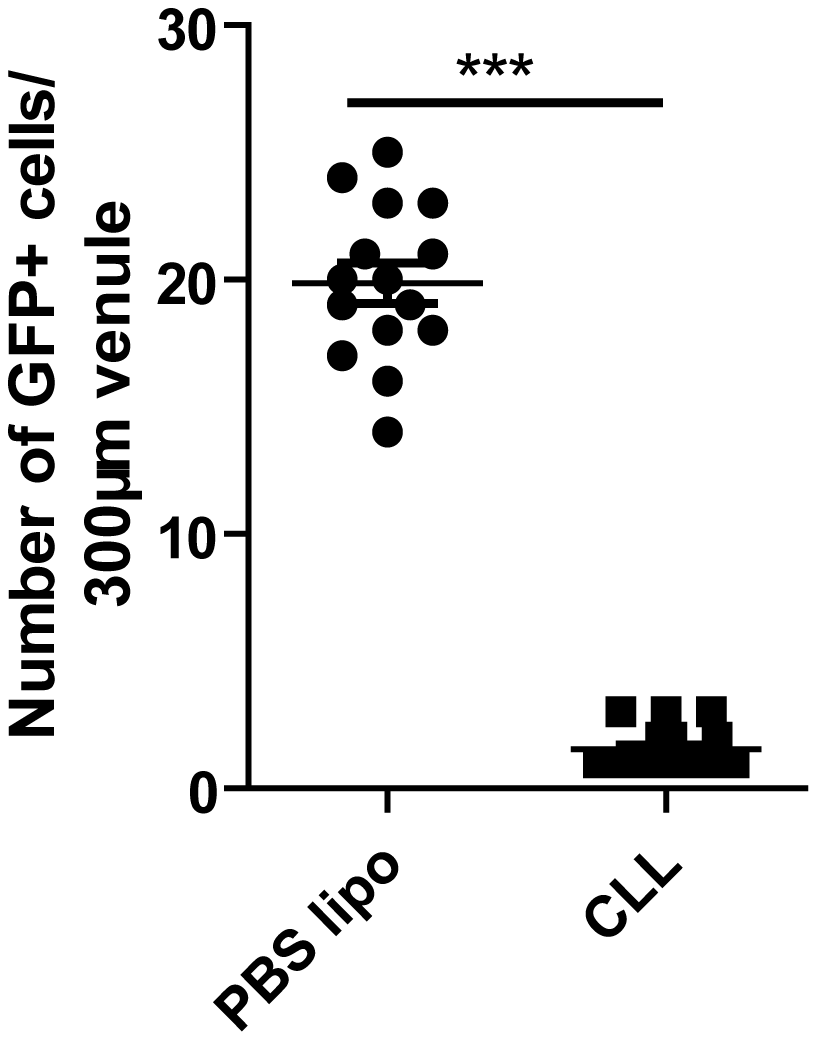

Supplement: S2 Fig — CX3CR1gfp/+ mice (n = 5 per group) were i.v. administered with 200 μl Clodronate liposomes (CLL) to deplete monocytes or PBS liposomes as control. The mice were i.v. infected with 20x106 C. neoformans H99 24 h later. IVM was performed on the brain 24 h post infection to enumerate GFP+ cells recruited to the brain postcapillary venules. Data are expressed as mean ± SEM. ***, p<0.001 by two-tailed student’s t test. (TIF) [file ppat.1008361.s002.tif]

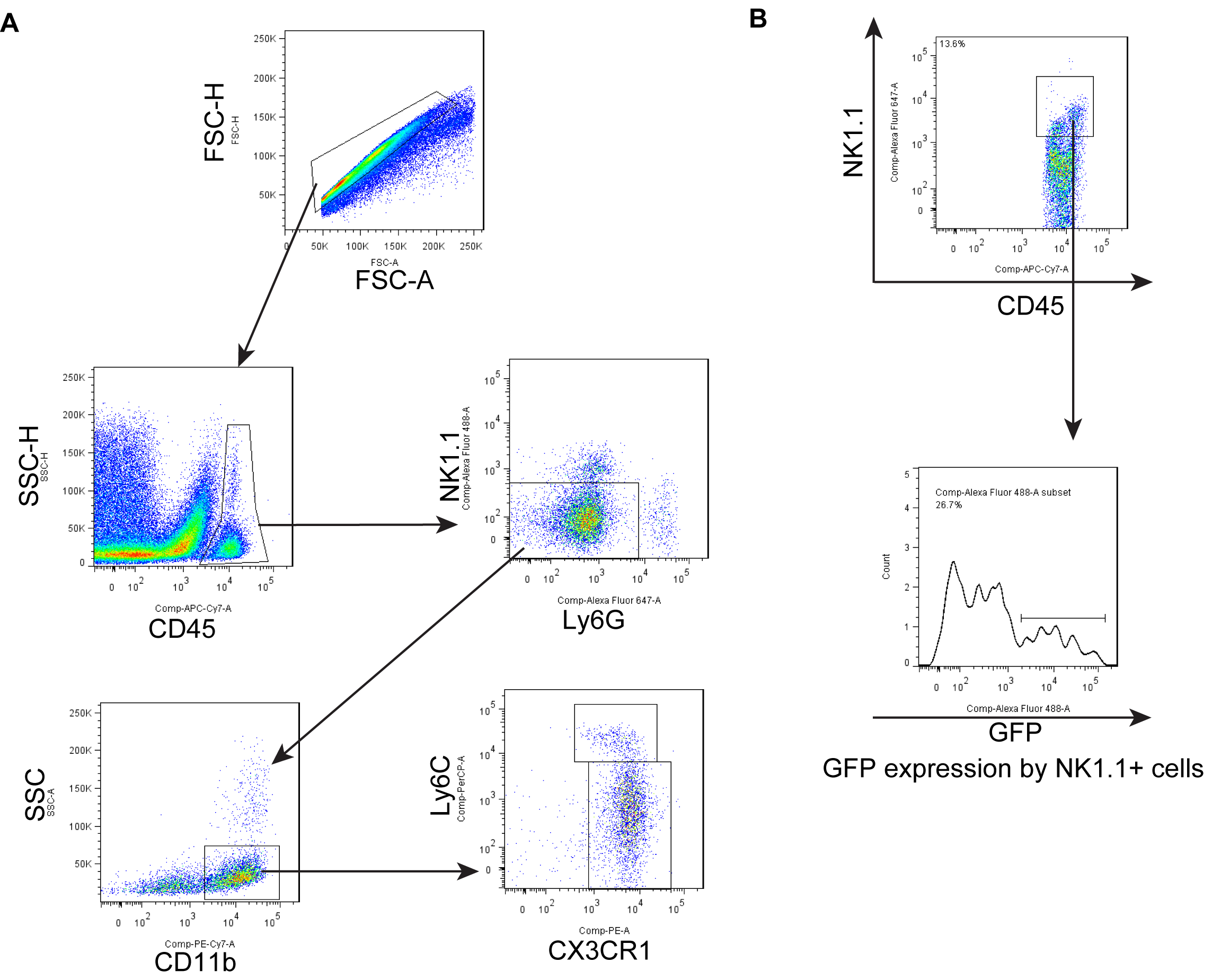

Supplement: S3 Fig — (A) Ly6Chi monocytes were defined as CD45+Ly6G-NK1.1-CD11b+CX3CR1+Ly6Chi, while Ly6Clow monocytes as CD45+Ly6G-NK1.1-CD11b+CX3CR1+Ly6Clow. Microglia express intermediate level of CD45; they were gated out after selecting CD45+ population. Leukocytes were isolated from the brain of C57BL/6 mice 24 h after i.v. infection with 20x106 C. neoformans. (B) A representative flow cytometry histogram showing the expression of GFP by CD45+NK1.1+ cells. CX3CR1gfp/+ mice were i.v. infected with 20x106 C. neoformans. 24 h later, leukocytes were purified from the brain of infected mice for flow cytometry analysis. (TIF) [file ppat.1008361.s003.tif]

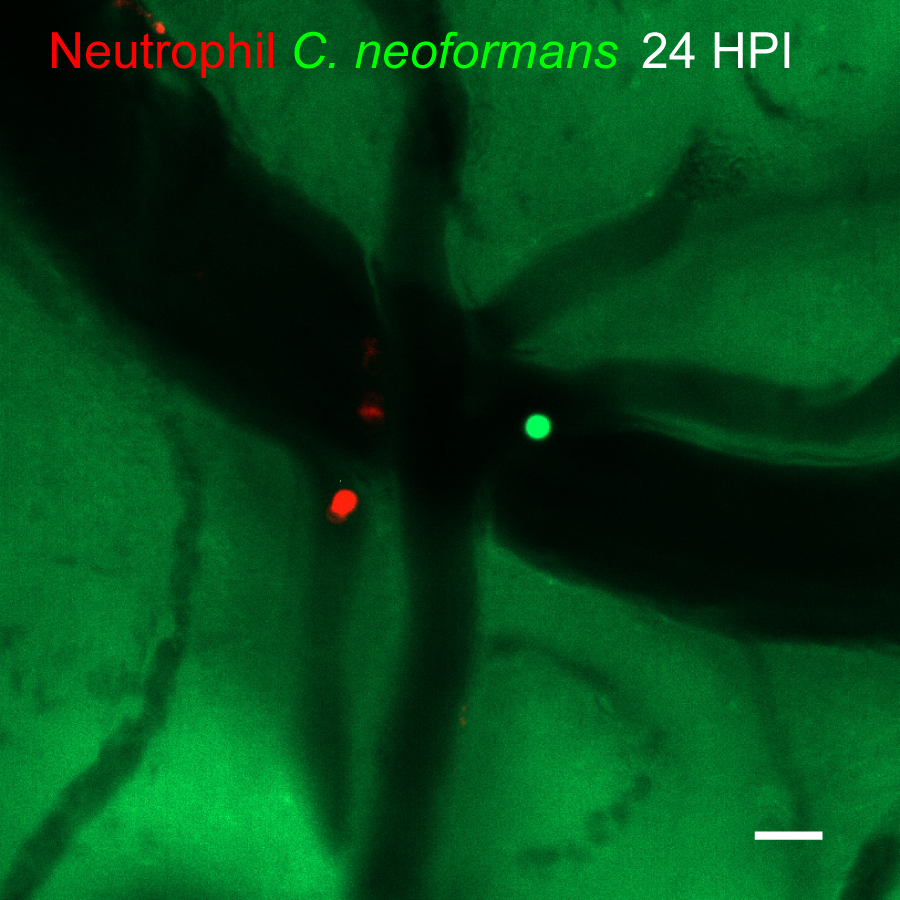

Supplement: S4 Fig — C57BL/6 mice were i.v. infected with 20x106 GFP-labeled C. neoformans. A representative IVM image showing neutrophils in the brain of infected mice 24 h after infection. The mice were i.v. injected with 2 μg AF647-anti-Ly6G mAb to label neutrophils 5 min before imaging. C. neoformans: green, neutrophils: red. Scale bar: 10 μm. (TIF) [file ppat.1008361.s004.tif]

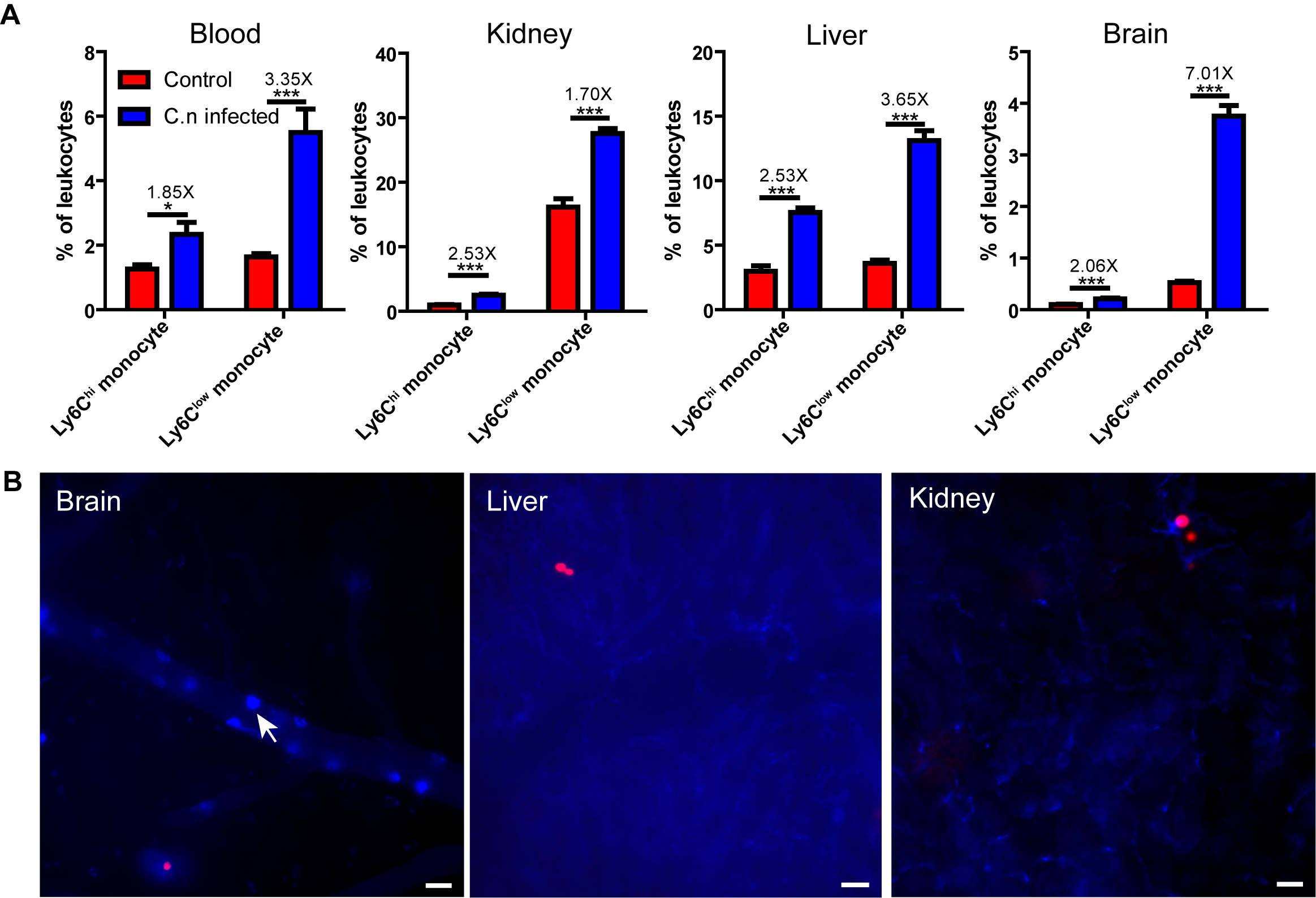

Supplement: S5 Fig — (A) Mice (n = 5 per group) were i.v. infected with 20x106 C. neoformans H99. Twenty-four hours later, different organs were collected for enumeration of monocytes (Ly6Chi monocytes: CD45+CD11b+CX3CR1+Ly6Chi; Ly6Clow monocytes: CD45+CD11b+CX3CR1+Ly6Clow) by flow cytometry. (B) Representative IVM images showing the recruitment of CX3CR1+ monocytes 24 h after i.v. infection with 20x106 C. neoformans H99 (red). Mice were i.v. injected with 5 μg AF647 conjugated anti-CX3CR1 mAb 10 min before imaging to label monocytes (blue). Scale bar 20 μm. Data expressed as mean ± SEM are representative of 2 independent experiments. *, p<0.05; ***, p<0.001 by two-way ANOVA. (TIF) [file ppat.1008361.s005.tif]

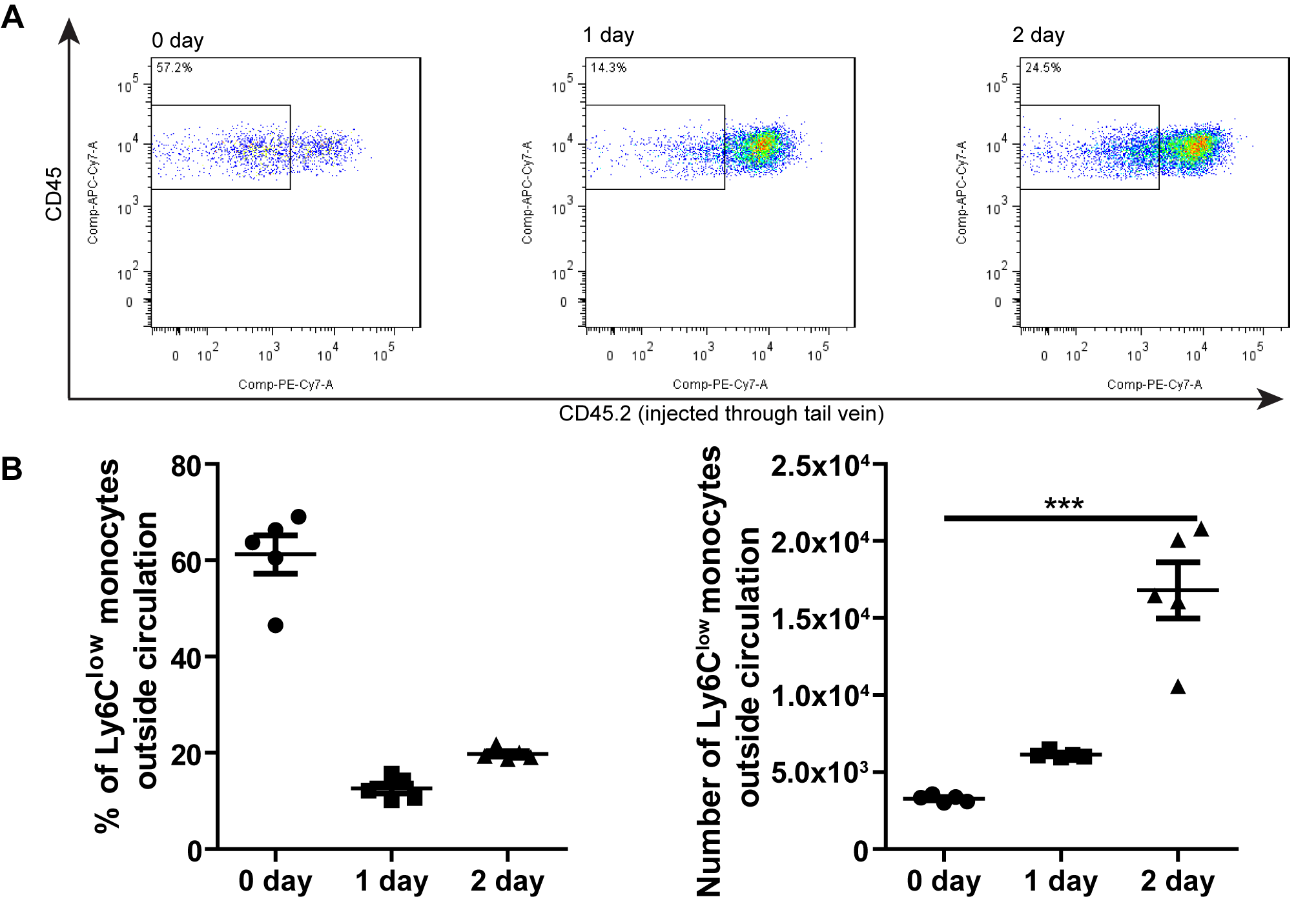

Supplement: S6 Fig — Mice (n = 5 per group) were i.v. infected with 20x106 C. neoformans H99 for 1 or 2 days. Ten minutes before euthanasia, mice were i.v. treated with 5 μg PE-Cy7 conjugated anti-CD45.2 mAb (clone: 104), which does not block CD45 (clone: 30-F11) mAb binding, to label all circulatory leukocytes. Brain leukocytes were then isolated and analyzed by flow cytometry. (A) Representative plots showing the percentage of transmigrated Ly6Clow monocytes (CD45.2-, outside brain blood vessels) out of the total brain CD45+ leukocytes. (B) The percentage (left) and number (right) of transmigrated Ly6Clow monocytes over the time. Data are expressed as mean ± SEM. ***, p<0.001 by one-way ANOVA followed by Tukey’s test. (TIF) [file ppat.1008361.s006.tif]

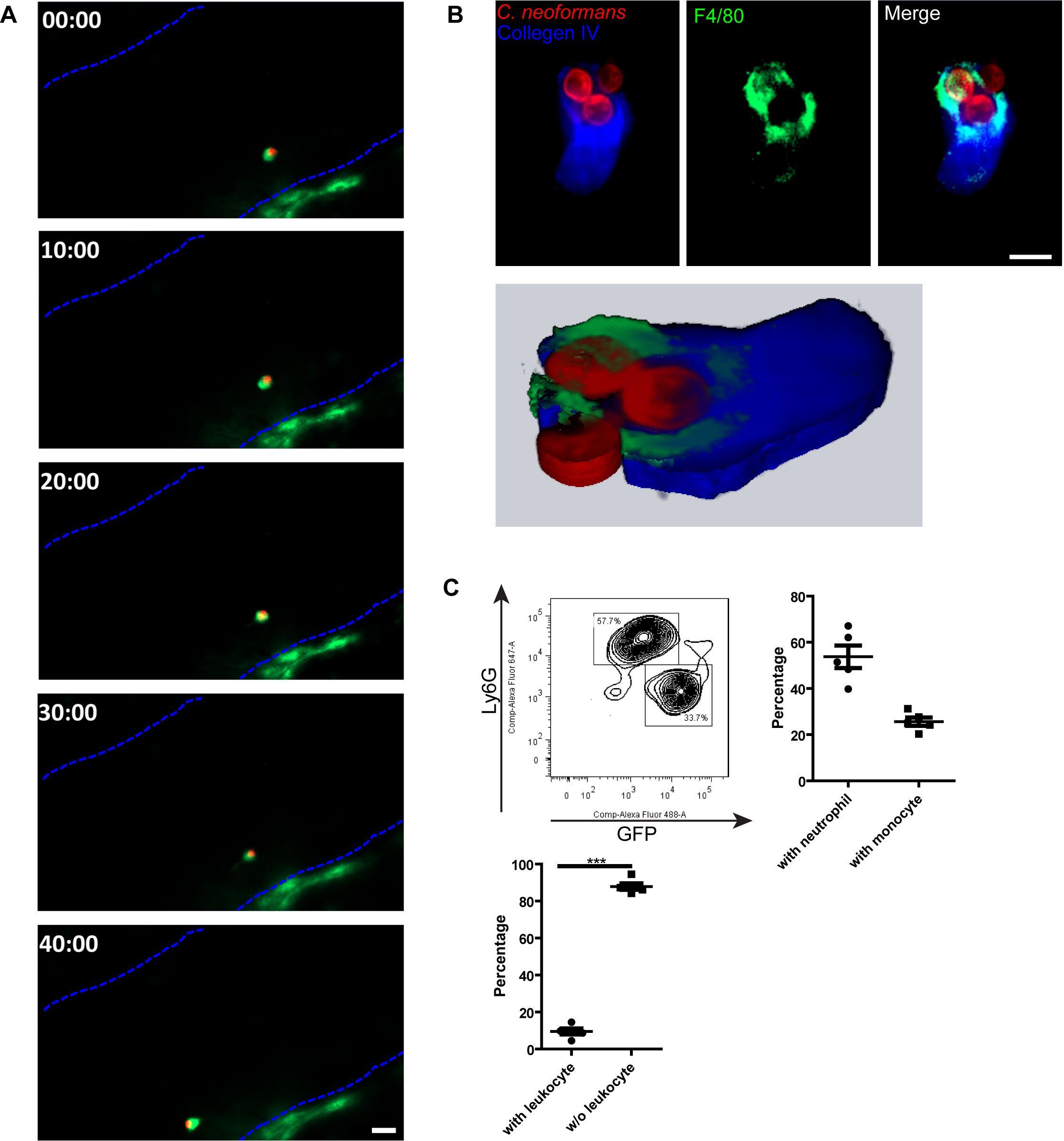

Supplement: S7 Fig — (A) A series of IVM images showing that a GFP+ monocyte (green) carrying C. neoformans (red) was crawling in the luminal side of a postcapillary venule of CX3CR1gfp/+ mice 18 h post i.v. infection with 20x106 C. neoformans. (B) Immunohistochemistry showing a monocyte containing multiple C. neoformans, one of which appeared to spread from the monocyte to an endothelial cell. C57BL/6 mice were infected with 20x106 C. neoformans and euthanized 18 h after infection for immunohistochemistry. Upper panel: 2D images; lower panel: 3D image. Monocytes: green, C. neoformans: red, vessel: blue. (C) The percentages of phagocytes carrying C. neoformans. CX3CR1gfp/+ mice (n = 5) were infected with 20x106 Uvitex 2B labeled C. neoformans. Brain leukocytes were purified 18 h post infection for flow cytometry analysis. Initially, CD45+Uvitex 2B+ population were gated. The percentages of monocytes carrying C. neoformans (Ly6G-GFP+) and neutrophils carrying C. neoformans (Ly6G+GFP-) were analyzed. Upper left panel: a representative plot, upper right panel: quantification, lower panel: the percentage of free yeast cells in the brain. Scale bars: 10 μm. Data are expressed as mean ± SEM and representative of 2 independent experiments. ***, p<0.001 by two-tailed student’s t test. (TIF) [file ppat.1008361.s007.tif]

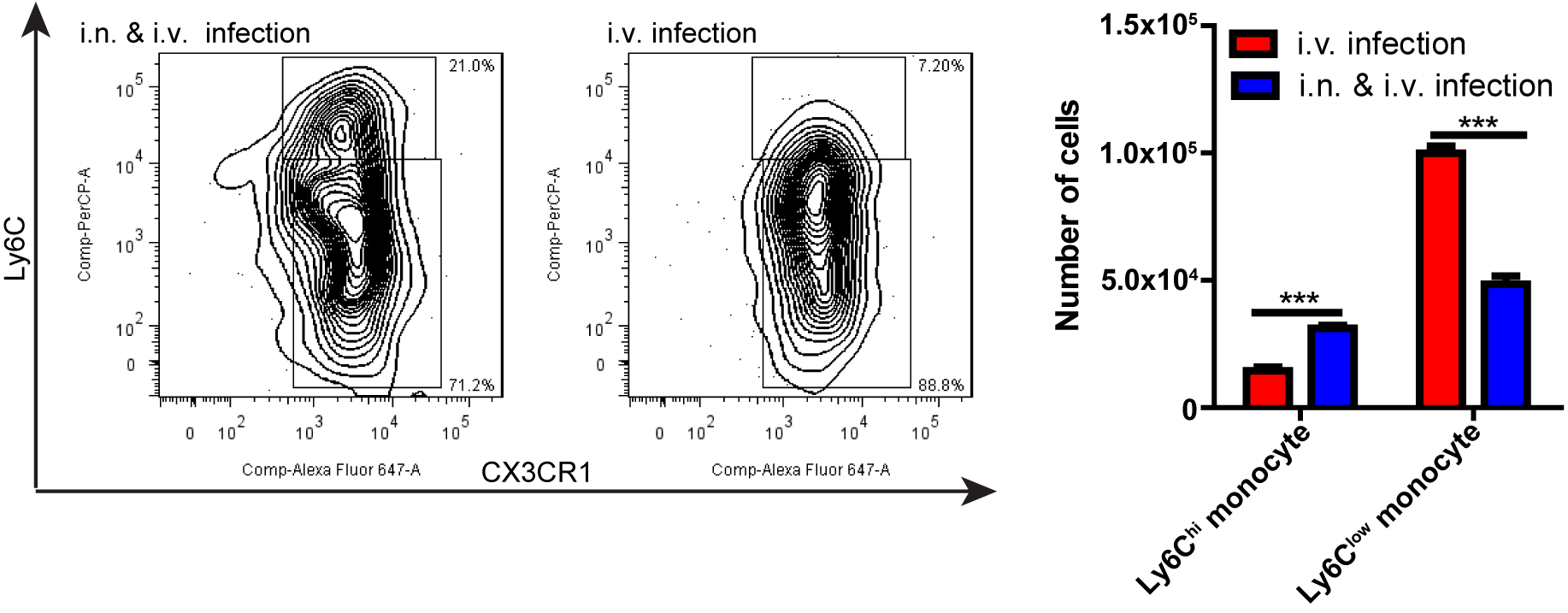

Supplement: S8 Fig — Mice (n = 5 per group) were intranasally (i.n.) infected with 1x10^4^ C. neoformans H99. Two weeks later, the infected mice (i.n. & i.v. infection group) and uninfected control mice (i.v. infection group) were infected with 20x106 C. neoformans H99 through the tail vein. Twenty-four hours later, Ly6Chi and Ly6Clow monocytes in the brain were analyzed by flow cytometry. Left: representative plots showing Ly6Chi and Ly6Clow monocytes. Right: quantification of Ly6Chi and Ly6Clow monocytes. Data expressed as mean ± SEM are representative of 2 independent experiments. ***, p<0.001 by two-way ANOVA. (TIF) [file ppat.1008361.s008.tif]

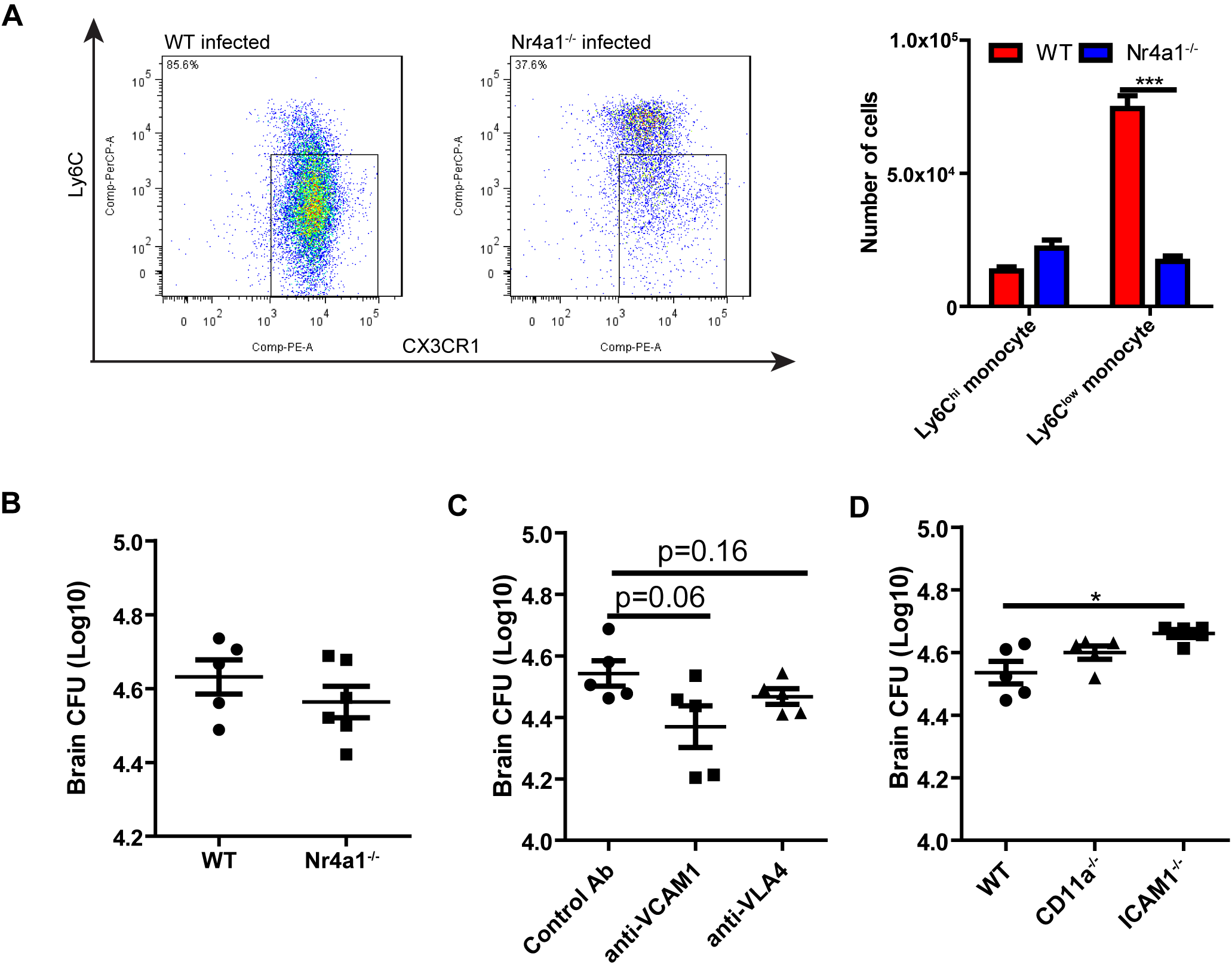

Supplement: S9 Fig — (A) WT mice and Nr4a1-/- mice (n = 5 per group) were i.v. infected with 20x106 C. neoformans H99 for 24 h; brain monocytes were analyzed by flow cytometry. Left: representative plots showing Ly6Chi and Ly6Clow monocytes in the brain; right: the number of Ly6Chi and Ly6Clow monocytes in the brain. (B) Brain fungal burdens of WT and Nr4a1-/- mice (n = 5–6 per group) 48 h after i.v. infection with 5x10^4^ C. neoformans H99. (C) Brain fungal burdens of WT mice 48 h after i.v. infection with 5x10^4^ C. neoformans H99. Mice (n = 5 per group) were treated with 100 μg anti-VCAM1, anti-VLA4 antibody or control antibody on day 0. (D) Brain fungal burdens of WT, CD11a-/-, ICAM1-/- (n = 5 per group) 48 h after i.v. infection with 5x10^4^ C. neoformans H99. Data are expressed as mean ± SEM. *, p<0.05; ***, p<0.001 by two-tailed student’s t test. (TIF) [file ppat.1008361.s009.tif]

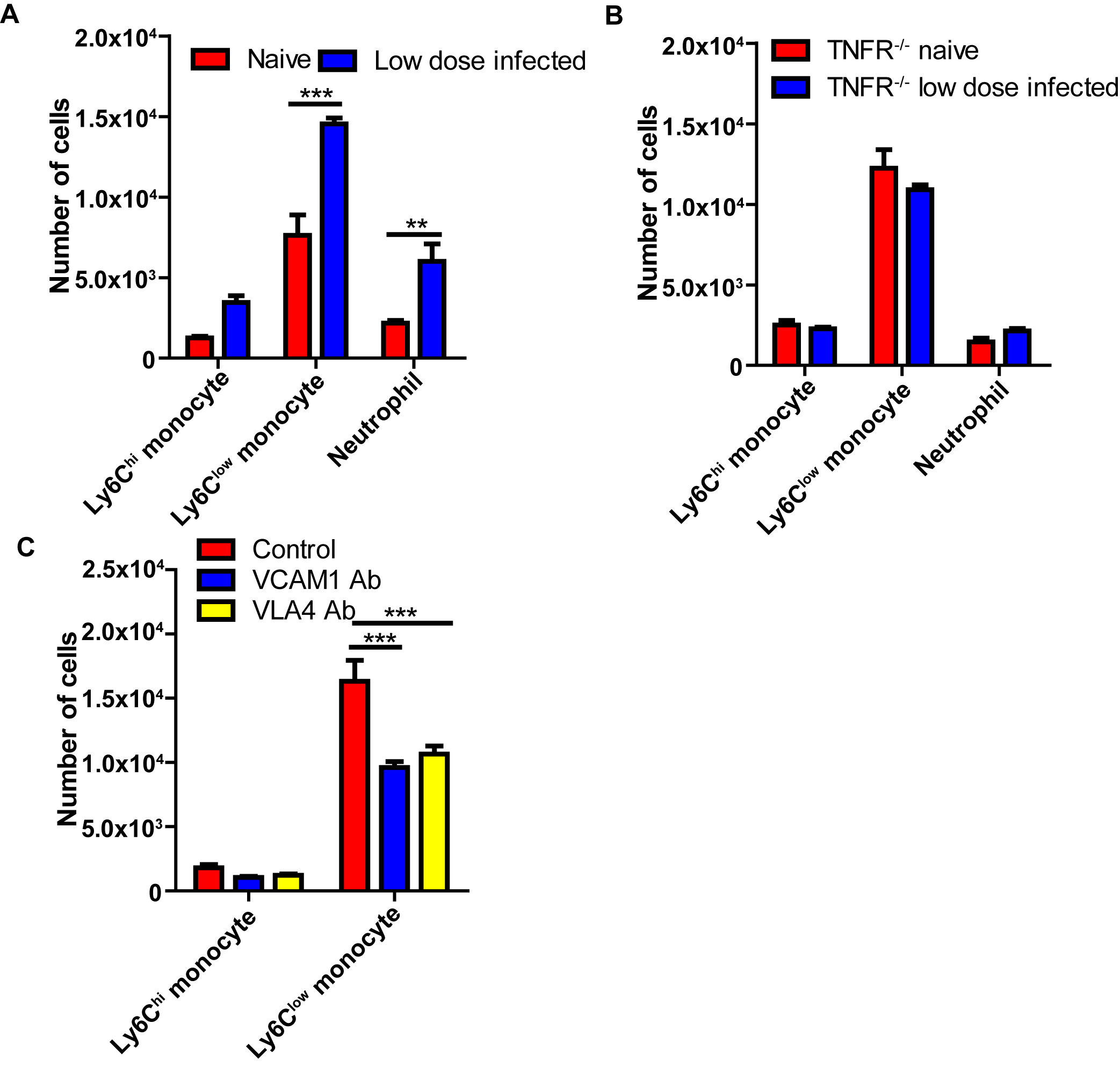

Supplement: S10 Fig — (A) Wild-type mice (n = 5 per group) were i.v. infected with 5x10^4^ C. neoformans H99. Twenty-four hours later, infected and naïve mice were euthanized and the numbers of leukocytes in the brain were enumerated by flow cytometry. (B) TNFR-/- mice (n = 5 per group) were i.v. infected with 5x10^4^ C. neoformans H99. The numbers of leukocytes in the brain of infected mice and naïve mice were counted 24 h after infection by flow cytometry. (C) Wild-type mice (n = 5 per group) were i.v. infected with 5x10^4^ C. neoformans H99 for 24 h. 20 min before euthanasia, mice were treated with anti-VCAM1 or anti-VLA4 mAb. The numbers of Ly6Chi and Ly6Clow monocytes were enumerated by flow cytometry. Data are expressed as mean ± SEM. **, p<0.01; ***, p<0.001 by two-way ANOVA. (TIF) [file ppat.1008361.s010.tif]
