# Supplementary material for: VCAM1/VLA4 interaction mediates Ly6Clow monocyte recruitment to the brain in a TNFR signaling dependent manner during fungal infection
Source: PLoS Pathog. 2020 Feb 26;16(2):e1008361. doi: 10.1371/journal.ppat.1008361 (PMC7062284; doi:10.1371/journal.ppat.1008361)
Supplement: Attachment — Submitted filename: Response to reviewers.docx [file ppat.1008361.s022.docx]

Responses to the reviewers

Editorial Board PLOS Pathogens

Dear Editor,

We would like to thank you for the opportunity to revise and resubmit our manuscript entitled “VCAM1/VLA4 interaction mediates Ly6C^low^ monocyte recruitment to the brain in a TNFR signaling dependent manner during fungal infection”. We are grateful to the reviewers for their very thoughtful comments, which have allowed us to significantly improve the manuscript. We carefully considered the reviewer’s comments and performed new experiments to address the reviewer’s concerns. We apologize for the delay in the submission of the revised version due to breeding various types of knockout mice and performing a few novel experiments suggested by the reviewers.

*Reviewer's Responses to Questions*

***Part I - Summary*** *Please use this section to discuss strengths/weaknesses of study, novelty/significance, general execution and scholarship.

Reviewer #1: This intravital microscopy study, combined with analysis of CNS immune response responses focuses on the of Ly6Clow monocytes during the early stages of CNS disseminating fungal infection caused by C. neoformans (C.n). Study demonstrates rapid/early recruitment to the brain vasculature (within 12-24h) of monocytes, chiefly LY6Clow subset, following intravenous inoculation with a high-dose of C.n. H99. The recruitment depends primarily on the interaction of VCAM1 expressed on the brain endothelium with VLA4 expressed on Ly6Clow monocytes. TNFR signaling is essential for the recruitment through enhancing VLA4 expression on Ly6Clow monocytes. Some of these Ly6Clow monocytes contain internalized C. neoformans and carried the organism while crawling on and adhering to the luminal wall of brain vasculature and migrating to the brain parenchyma. This study highlights important involvement of this subset during infection. While focus on the Ly6Clow monocytes in the context of disseminated C.n., followed by CNS infection is novel and interesting and could potentially be an important advance, there are some significant concerns:
First, the presented results are difficult to reconcile with previous studies showing that CCR2/CCL2 axis is involved in human patients with CNS C.n. infection (Jarvis et all 2015; Panackal et al 2015) and CD11b+Ly6C+ myeloid cells are recruited in high quantities into the brain of mice with CNS C.n. infection (Neal at al 2017). These studies have not been cited or discussed by the authors. However, these published data STRONGLY argue against proposed here "unique link" of Ly6Clow monocytes with cryptococcal brain infection, as opposed to other organs with C.n-infection or the CNS infections with other microbes.* *The more likely reason for predominance of Ly6Clow monocytes in this model is that the relatively early time points (1-3 days) are used prior to CCR2+ monocyte BM mobilization, and the known delay in recruitment of Ly6Chi/CCR2+ monocytes, typically past day 7 and day 14 in the C.n-infected lungs or CNS, respectively.
Second, it is not clear what fraction of these intravascular monocytes actually crosses to the brain (as implied by the title and several paragraph titles). Thus, at this point, it is hard to understand relative importance of these monocyte subsets in host defenses/pathogenesis of the C.n-induced CNS disease without any quantitative data in this respect.*

We agree with the reviewer and thank for his/her comments. We cited those important published work and added discussion in the revised version (Line 284-292). We also performed new experiments to address the reviewer’s concerns (see below). We show that, at early time point of infection, more Ly6C^low^ monocytes but relatively less Ly6C^hi^ monocytes were recruited to the brain. Published literature showing that Ly6C^hi^ monocytes were recruited to the brain at a later timepoint (two weeks after infection) but not at the early time point of infection (Neal et al 2017, mBio). Therefore, our finding is not controversy to previous findings, instead, it is complementary (or adding new information) to previous findings. In contrast to previous studies, our studies focus on the dynamics of monocyte recruitment to the brain and the dynamic interactions of monocytes with fungal pathogens in the brain by taking advantage of intravital microscopy technique, a topic that has not been well studied not only for fungal infections but also for bacterial and parasitic infections. *Reviewer #2: Overall, the studies were well executed and the findings/interpretations are supported by the data presented. Additionally, the article is well-written. The strengths of the study is that the authors findings were supported using multiple animal models and good visuals. Nonetheless, a weakness of the study is a concern that the infectious dose combined with the route of inoculation used in the study (20 x 10^6 i.v.) is so over-bearing on the immune response that the resulting observations are not relatable clinically. The overall consensus is that infection begins via intranasal inhalation and that the organisms may eventually disseminate to the CNS (typically in an immune suppressed host but not entirely). This allows for the development of a much different type of immune response and possibly more time for monocytes to differentiate into various other cell types such as DCs. Thus, the findings presented in the manuscript may only be a reflection of the model system. This may need to be addressed to put the observations in context or some additional studies done using a much smaller infectious dose and/or route of infection.*

We agree with the reviewer. The goal of the study is to examine the dynamics of monocyte recruitment to the brain during fungal infection and its dynamic interaction with fungal pathogens. To address these questions, we need to use a dynamic observation approach such as intravital microscopy. As every technique, intravital microscopy has both advantages and disadvantages. To ensure visualization, we need to intravenously inject a great number of yeast cells. We realized the limitation of our model system and performed new experiments with a lower infectious dose using flow cytometry as suggested by the reviewer (see below). We also performed an experiment with both intranasal and intravenous infections as suggested by the first reviewer (see below).

*Reviewer #3: This is a study by Sun et al that defines the kinetics of accumulation of Ly6c low monocytes in the brain of Cryptococcus-infected mice and examines molecular factors that are associated with these cells' recruitment into the tissue. The authors utilize intravital microscopy to visualize some of these events and show that VCAM1/VLA4 and TNF receptor signaling is important for Ly6c low accumulation in the context of cryptococcal meningitis. This is a potentially interesting topic, with description of a cell type that has not been examined thoroughly during infections, and particularly during fungal infections. The authors carefully perform experiments to outline the molecules that may be critical for cell accumulation in the fungal-infected brain. The paper is well-written. However, the manuscript lacks 2-3 very important pieces of information to provide it with the significance in advancing the field as outlined below.*

We agree with the reviewers. New experiments were performed accordingly (see below).

***Part II – Major Issues: Key Experiments Required for Acceptance*** *Please use this section to detail the key new experiments or modifications of existing experiments that should be absolutely required to validate study conclusions.

Generally, there should be no more than 3 such required experiments or major modifications for a "Major Revision" recommendation. If more than 3 experiments are necessary to validate the study conclusions, then you are encouraged to recommend "Reject".*

***Reviewer #1****: The key experiment to address the first concern would be to monitor the vasculature of other organs (e.g. kidney and liver) in this C.n high-dose IV bolus disseminated infection model, to determine if similar microvasculature recruitment of Ly6Clow versus Ly6Chigh monocytes will be observed outside of the CNS in other organs during fungemia.*

As suggested, we evaluated the increase of monocytes in the brain, liver and kidney following infection in comparison to the blood using flow cytometry and intravital microscopy. As shown in the new Figure S5 and described in the manuscript (Line 161-163), the increase of Ly6C^low^ monocytes in the brain is dramatic compared to the blood. In contrast, the increase of Ly6C^low^ monocytes in the liver and kidney is comparable or less to the increase in the blood. These results suggested that the brain is unique for accumulation of Ly6C^low^ monocytes in this C.n disseminated infection model.

*It would be also worthwhile to induce an inhalation C.n infection 1-2 week prior to the IV-bolus infection to activate bone marrow to produce both subsets of monocytes first, and then follow with the IV infection/microscopy studies. Such model would be more consistent with natural history of cryptococcal infection, when the infection initially develops in the lungs, triggers some level of host response, but then disseminates into the CNS. This approach would allow to verify the relative contribution of each monocyte subsets in the CNS in a more "natural history disease setting" and allow for better comparisons with other studies (as they all focus on less acute time points).*

As suggested, we induced an inhalation C.n infection 2 weeks before i.v infection. The results showed that initial pulmonary infection enhanced brain accumulation of Ly6C^hi^ monocytes, associated with reduced recruitment of Ly6C^low^ monocytes in the brain (Line 290-292; Figure S8). It is likely that it takes time for mobilization of Ly6C^hi^ monocytes from the bone morrow to the brain, which is consistent with recent publication by Neal *et al* (2017, mBio).

*The second major concern regarding true monocyte recruitment to the brain could be addressed using short-term injection with fluorescent antibody or other monocyte tracer IV infusion (such as pkH20) at the onset of monocyte vascular accumulation and compare with similar approach at day 3 after infection and flow cytometry study to determine what fractions of Ly6C low monocles remained in the vasculature (tracer positive) and what fraction crossed BBB (tracer negative) over time. This study would help to clarify the claim of monocyte recruitment to the brain (as stated in the title of the manuscript).*

*If authors cannot produce the requested data the conclusions should be significantly toned down, limitations of the study acknowledged, and alternative interpretations of their data should be provided.*

As suggested, we used a fluorescent mAb to label leukocytes in the blood stream to distinguish those in the parenchyma. The result showed an increase of monocytes crossing the BBB over time. These results confirmed that this subset of monocytes migrates into the brain parenchyma (Line 163-164; Figure S6).

***Reviewer #2:*** *1. The authors should perform studies using a much lower infectious dose via the i.v. to show the necessity of VCAM1/VLA4 interactions and TNFR signaling on the recruitment of Ly6C lo monocytes to the brain. Doing these studies via the pulmonary route of infection may not be feasible using this strain of C. neoformans (H99) b/c mice typically succumb to infection via asphyxiation.*

The reason why we used a high infectious dose is that we aimed to visualize the dynamics of monocyte recruitment and the dynamic interactions of monocytes with *C. neoformans* in the brain vasculature using intravital microscopy. To be consistent with intravital microscopy, we also use the same dose for flow cytometry analysis. We agree with the reviewer and returned to the lab and performed the experiments with a much lower (5x10^4^) infectious dose. As shown in Figure S10 and described in Line 371-372, the results confirmed the observations in mice infected with a high infections dose.

***Reviewer #3:*** *1. Although the authors describe accumulation of Ly6c low monocytes in the fungal-infected brain and define some molecules important for this process, there is absolutely no information with regard to whether Ly6c low monocytes are protective or detrimental during infection or whether their accumulation plays no role. Therefore, the authors should perform two sets of experiments to enlighten the readers on this very important topic:
1a- Is Cryptococcus fungal load greater in any of the models that the ly6c low monocyte accumulation is low? That is in mice with impaired VCAM1 or VLA4 or CD11a or ICAM1?*

*1b- Is Cryptococcus fungal burden increased in* *Nr4a1-deficient mice that have been reported to specifically lack Ly6c low monocytes? (Carlin et al., Cell, 2013; Thomas et al., Immunity, 2016).*

*These 2 sets of experiments are in this reviewer's opinion critical to establish a functional role of Ly6c low accumulation in this model. Without that, it is unclear what the significance of modest decreases of Ly6c low monocytes in this model mean.*

We appreciate the reviewer for the excellent points. We returned to the lab and performed the experiments suggested by the reviewer. Infected ICAM1^-/-^ mice had higher brain CFU (Figure S9D and Line 358-359), which may reflect less killing of *C. neoformans* in the brain due to reduced recruitment of neutrophils, because that ICAM1 is also required for neutrophil recruitment and that neutrophils have been shown to kill *C. neoformans*.

We repeatedly observed a slight reduction of brain fungal burdens in the brain of infected mice treated with anti-VCAM1 or anti-VLA4 (Figure S9C; Line 356-357). We also repeatedly observed a tendency of reduction of brain CFU in infected Nr4a1^-/-^ mice compared to infected wild-type mice (Figure S9B; Line 355-356). However, the reduction did not reach statistical significance. These results complicate our interpretation of the contribution of Ly6C^-^ monocytes to *C. neoformans* invasion into the brain. In vitro and/or in vivo evidence has been previously provided to show that *C. neoformans* can use multiple mechanisms including transcytosis, paracytosis, and Trojan horse (phagocytes) to invade the brain. Although Ly6C^-^ monocytes were seen to interact with *C. neoformans* and carry this organism while crawling on and adhering to vessel and migrating to the brain, it is likely that Ly6C^-^ monocytes make less contribution to *C. neoformans* brain invasion compared to other mechanisms in our model system. It is also possible that the contribution of Ly6C^-^ monocytes to fungal brain invasion was overridden by other mechanisms in our model system. Since brain invasion by *C. neoformans* involves multiple mechanisms, further studies are definitively required to determine the relative contribution of Ly6C^-^ monocytes compared to other mechanisms involved in fungal brain invasion. We believe that comparison of the relative contribution of Ly6C^-^ monocytes to other mechanisms is beyond the scope of the current study. However, we have carefully discussed this point in the section of discussion (Line 359-366).

*2. In Figure 5C, are the authors sure that the CD31+ cells are indeed endothelial cells and that the staining is not an artifact? Have they used CD102 to also stain? FACS sorted the cells to confirm by pPCR or WB that indeed these are endothelial cells? Brain preparations don't typically contain many endothelial cells unless specific protocols are followed, and such protocols are not specified in the methods by the authors. To extend this, the data on TNFR expression by these CD31+ cells in Figure 5E is quite unimpressive. The authors should use a positive control Ab that is known to be expressed in endothelial cells to prove that these are indeed valid data*

To address the reviewer’s concern, we checked CD102 expression on the CD45^-^CD31^+^ population using two colors AF488 (showed) and AF647. As compared with the relative isotype control, this population expresses CD102, thus confirming their endothelial lineage (Figure 5D). We also confirmed that the cells in Figure 5E are endothelial cells using the new gating strategy.

*3. In Figure 5G the authors "normalize" numbers of Ly6c low monocytes relative to microglia. This is not the correct way to do this analysis. Absolute numbers of Ly6c low monocytes is important to show. In addition, these chimera data are suggestive but not proving of the point. Competitive repopulation studies of Ly6clow monocytes from 50% WT and 50% TNFR KO mice (1:1 mix, using different congenic markers) is the way to show that there is differential recruitment/accumulation. Therefore, the authors should not "normalize" numbers of monocytes to microglia and they should perform a competitive repopulation study to conclusively examine their hypothesis.*

We agree with the reviewer. We could not find suitable congenic markers for monocytes. We tried CD90.1 and CD90.2, however, these markers are not expressed on monocytes. Alternatively, we labeled monocytes with two different lipophilic dyes, PKH26 and CellVue Claret. We repeated the experiments using this new approach and replaced the original figures (Figure 5G; Line 229-231).

***Part III – Minor Issues: Editorial and Data Presentation Modifications*** *Please use this section for editorial suggestions as well as relatively minor modifications of existing data that would enhance clarity.*

***Reviewer #1:*** *Ln 91-92: the 600,000 numbers come from the older reports from the peak years of infections. Present estimate is around 180,000 deaths per year. please use more current reference such as the recent colloquium report of Academy of ASM*

We updated the reference and corrected the numbers accordingly.

Ln 108-9 "following brain infection": to be precise the study is not restricted to brain infection but follows the acute fungemia, a model of acutely disseminated C.n. infection.

We changed it to “using a model of acutely disseminated *C. neoformans* infection”

*Ln: 117 the paragraph title is somewhat misleading since it is not clear from the study if the monocytes were recruited to the brain, brain vasculature or the vasculature of all other tissues*

We changed it to “to the brain vasculature”

*LN 138 In the absence of additional data it is not clear which subset of monocytes predominantly was recruited/crossed to the brain*

We provided additional data which showed the migration of Ly6C^low^ monocyte to the brain parenchyma (Figure S6, Line 163-164).

*Other titles Ln 167, 189, 206 should be probably changed from "to the brain" to "to the brain vasculature", as most images seem to show monocytes in the vasculature.*

We changed it to “to the brain vasculature”.

*Since microglia express CX3CR1, the author should how they gate out microglia in their flow cytometry analysis such as Fig 2C. Does the CXCR3+Ly6Clow gate also contain microglia if you initially gated on CD45+ cells?*

No, microglia were not included in the population. Microglia only express low levels of CD45, and all other leukocytes have high levels of CD45. This is a very clear difference by flow cytometry. Since we gated on all CD45^+^ leukocytes, microglia will be excluded. We added that statement to the legend of Figure S3.

*It is somewhat arbitrary what is "massive" cell recruitment to the brain (LN 40, 117, 136, 341, 661, 663, 848) but total cell numbers reported here are not that overwhelming in numbers. Perhaps this could be toned down to a "substantial".*

We agree and changed it to “substantial” throughout the manuscript.

***Reviewer #2:*** *1. It is not clear by the monocyte population is compared to microglia (Figure 5G).*

As reviewer 3 also suggested, we repeated the experiment using competitive repopulation study by injecting WT and TNFR^-/-^ monocytes in a 1:1 ratio. The total number of donor monocytes are now shown (Figure 5G, Line 229-231).

*2. Do the authors suspect a difference in morbidity/mortality will be observed by blocking VCAM1/VLA4 interactions and/or TNFR signaling (perhaps due to less inflammatory cells trafficking to the brain)?*

We saw a slight reduction in the brain fungal burden after blocking VCAM1/VLA4 (Figure S9). We did not see mortality difference as the H99 stain can proliferate rapidly in the brain and kill the mice rapidly.

*3. Adhesion misspelled in Y axis of Figure 4C.*

We corrected it in the revision.

***Reviewer #3:*** *1. Why did the authors use VLA4 and VCAM1 Abs and not genetic deletion of these molecules? Are these mice not available?*

As described on Jackson lab website, VLA4 and VCAM1 knockout mice exhibit embryonic lethality (“<https://www.jax.org/strain/002463>” and “<https://www.jax.org/strain/002992>”), thus both mouse strains are not available for the moment. By the way, blocking antibodies have been extensively used to study the functions of VLA4 and VCAM1 in leukocyte recruitments.

*2. Please show CD11b in Figure 2C. Are all CX3CR1+ cells (Ly6c hi and low) all CD11b+?*

Yes, they both express high levels of CD11b, see new Figure 2C.

*3. Please label Figure 6 appropriately (is #7 now)*

We corrected it in the revision.

PLOS authors have the option to publish the peer review history of their article ([what does this mean?](https://journals.plos.org/plospathogens/s/editorial-and-peer-review-process#loc-peer-review-history)). If published, this will include your full peer review and any attached files.


If you choose “no”, your identity will remain anonymous but your review may still be made public.


**Do you want your identity to be public for this peer review?** For information about this choice, including consent withdrawal, please see our [Privacy Policy](https://www.plos.org/privacy-policy).

Reviewer #1: No

Reviewer #2: No

Reviewer #3: No
